# Surgery paradigm for locally advanced breast cancer following neoadjuvant systemic therapy

**DOI:** 10.3389/fsurg.2024.1410127

**Published:** 2024-09-06

**Authors:** Ziyue Sun, Kexin Liu, Yanru Guo, Nanyuan Jiang, Meina Ye

**Affiliations:** Department of Breast Surgery, Longhua Hospital, Shanghai University of Traditional Chinese Medicine, Shanghai, China

**Keywords:** locally advanced breast cancer, individualized treatment, neoadjuvant systemic therapy, surgery, pathological complete response

## Abstract

Locally advanced breast cancer (LABC) remains a significant clinical challenge, particularly in developing countries. While neoadjuvant systemic therapy (NST) has improved the pathological complete response (pCR) rates, particularly in HER2-positive and triple-negative breast cancer patients, surgical management post-NST continues to evolve. The feasibility of omitting surgery and the increasing consideration of breast-conserving surgery, immediate reconstruction in LABC patients are important areas of exploration. Accurate assessment of tumor response to NST through advanced imaging and minimally invasive biopsies remains pivotal, though challenges persist in reliably predicting pCR. Additionally, axillary lymph node management continues to evolve, with emerging strategies aiming to minimize the extent of surgery in patients who achieve nodal downstaging post-NST. Minimizing axillary lymph node dissection in favor of less invasive approaches is gaining attention, though further evidence is needed to establish its oncological safety. The potential for personalized treatment approaches, reducing surgical morbidity, and improving quality of life are key goals in managing LABC, while maintaining the priority of achieving favorable long-term outcomes.

## Introduction

LABC is commonly referred to inoperable cancers which surgical resection is impossible without systemic therapy and absence of distant metastases. In general, clinically stage III breast cancer was included (1, 2).

The implementation of a comprehensive breast cancer screening program has resulted in a comparatively low prevalence of LABC in developed nations (3). It still remains a big challenge in developing countries. For instance, in India, 47% of breast cancer cases are diagnosed at stage III (4). Despite the elevated risk of recurrence and metastasis, LABC can still be curable if local control is attained. Due to the use of dual human epidermal growth factor receptor 2 (HER2) blockade and platinum-based neoadjuvant chemotherapy, the rate of pCR rate in HER2 positive (HER2+) or triple-negative breast cancer (TNBC) patients has increased to more than 30% (5). Mastectomy and axillary lymph node dissection (ALND) are commonly employed as the standard surgical procedures for patients diagnosed with LABC. Surgery is performed with the objective of completely excising the primary tumor, as well as any adjacent skin or muscular involved. As the treatment approach for breast cancer transitions from “maximum tolerable” to “minimum effective” treatment, it is important to consider if there are additional surgical options available for patients with LABC, while considering the pretreatment stage and response to NST. This review seeks to investigate the potential for omitting breast surgery and the viability of breast-conserving surgery (BCS) or immediate reconstruction (IR) without compromising oncological safety for LABC. Additionally, as well as to identify targeted patients for ALND exemption, thus promoting individualized surgical options for LABC patients.

## Ways to evaluate the effectiveness of NST and possibility of omitting surgery

Assessing the response of breast cancer patients to NST before surgery is essential for tailoring personalized surgical plans and treatment strategies. In cases where patients achieve a clinical complete response (cCR), meaning no detectable cancer is found through physical examination and imaging, it may even be possible to consider omitting surgery (6). Magnetic resonance imaging (MRI) is more accurate in predicting pCR and residual disease compared to clinical examination, ultrasound and mammography (7, 8). However, MRI can either overestimate or underestimate residual disease. Overestimation may occur due to fibrosis, necrotic tumors, or residual benign masses, while underestimation can be caused by no mass lesions, invasive lobular carcinoma, hormone receptor-positive (HR+) tumors, nonconcentric shrinkage patterns, antiangiogenic therapy, or late-enhancing foci (9). Therefore, the accuracy of MRI is still falling short of clinical expectations. Moreover, the accuracy varies significantly across different molecular subtypes, with the highest sensitivity observed in TNBC and the lowest in HR+/HER2-subtypes (10). Therefore, relying solely on imaging results is insufficient. In recent years, multiple trials have explored the predictive value of image-guided minimally invasive biopsy (MIB) techniques, such as core needle biopsy (CNB), vacuum-assisted biopsy (VAB), and fine-needle aspiration (FNA), for determining breast pCR following NST. For example, the study by Sutton et al. (11) found that MRI-guided VAB can increase the accuracy of predicting pCR to 95%. However, Hemert et al. (12) found that small residual lesions (4–7 mm) are often tended to be missed in biopsy procedures. A meta-analysis (13) of nine trials involving 1,030 breast cancer patients found that, while the pooled sensitivity and specificity of MIB were 0.72 [95% confidence interval (CI): 0.61–0.81] and 0.99 [95% CI: 0.89–1.00], respectively, current image-guided MIB methods are still not accurate enough for reliably predicting breast pCR after NST.

The question of whether patients achieving cCR or pCR through MIB can be exempted from breast surgery has been addressed in previous retrospective studies by Ring (14) and Clouth (15), which included patients with stage III breast cancer. Their findings indicate that omitting breast surgery does not affect survival outcomes in the long run. However, there are no studies specifically examining the exemption of surgery in LABC. A multicenter phase II clinical trial (NCT02945579) (16) led by MD Anderson Cancer Center is exploring the possibility of omitting surgery after NST, but it has excluded LABC patients. Despite the pooled analysis published in *Lancet* (17) showing that patients who achieve pCR exhibit improved long-term survival rates, a recent meta-analysis (18) of 54 clinical studies found only a weak association between pCR and both disease-free survival (DFS) and overall survival (OS). The meta-analysis concluded that pCR should not be considered the primary endpoint in trials of NST for breast cancer. Currently, there are no effective methods available for accurately assessing pCR. Moreover, pCR cannot be considered a primary endpoint in research, as patients with LABC who achieve pCR are not yet exempt from surgery.

## Breast surgery

### Feasibility and safety of BCS in LABC patients

Is BCS considered a safe treatment option for LABC patients who exhibit positive response to NST? This paragraph explores the influence of tumor shrinkage patterns on the feasibility of BCS, evaluates the criteria for selecting patients for BCS, and discusses the implications of different margin definitions on clinical outcomes. The crucial aspect of BCS is to achieve a negative pathological margin, so it's important to understand the pattern of tumor regression. The tumor shrinkage patterns following NST predominantly exhibit concentric and non-concentric characteristics. Wang et al. (19) classified residual tumor morphology into three categories: isolated residual tumors (61%), multifocal and patchlike (33%), and main residues with satellite lesions (6%). Most tumors exhibited isolated concentric shrinkage, while the other two types demonstrated non-concentric shrinkage. The primary tumor's size directly influenced its concentric shrinkage pattern, with larger tumors more often showing non-concentric shrinkage, which complicates the attainment of negative margins. The application of BCS after NST is theoretically limited to tumors exhibiting concentric shrink patterns. For multiple lesions in the same quadrant, BCS can be attempted. The primary tumor in LABC is typically large and prone to be non-concentric. As a result, it is necessary to conduct imaging comparisons before and after NST in order to comprehensively assess the patterns of tumor shrinkage. Bi et al. (20) conducted a study on 3D MRI reconstruction of residual tumors, suggesting that a 50% reduction in the longest diameter and a size of ≤2 cm post-NST could qualify patients for BCS. This criterion could potentially expand the BCS-eligible patient population. After a median follow-up of 77 months, the rate of recurrence or metastasis was 7.1%. The National Comprehensive Cancer Network and St. Gallen consensus (21, 22) define negative margins for BCS after NST as “no ink on tumor,” consistent with criteria for BCS without NST. However, a 2022 meta-analysis in the *British Medical Journal* (23) challenged this standard, finding that close margins (defined as no tumor on ink but <2 mm) were linked to a higher risk of local recurrence and metastasis compared to negative margins (≥2 mm), even when accounting for adjuvant chemotherapy and radiotherapy (*P *< 0.001). This raises concerns about the adequacy of the “no tumor on ink” criterion for BCS post-NST. The safety of applying non-NST margin criteria to NST cases remains inconclusive due to insufficient high-level evidence.

The rate of BCS after NST in LABC patients ranged from 12.5% to 43.4% in several retrospective studies (24–27). BCS was found to be oncologically safe for LABC patients who responded well to NST. Younger patients, those with smaller tumors, and those achieving pCR were more frequently selected for BCS. Additionally, patients in the NST-BCS group were more commonly found to have HER2+/HR- or TNBC (24, 27), as well as non-invasive lobular carcinoma, compared to the mastectomy group (25, 26).

Sun et al. (28) conducted a meta-analysis of 16 studies, finding no significant difference in local recurrence-free survival (LRFS) between the BCS and mastectomy groups (*P* = 0.26). However, DFS and OS were higher in the BCS group (*P* < 0.01). This may be attributed to the higher pCR rates in the BCS group (29), which is associated with improved DFS and OS. While these results imply that BCS maybe safe for LABC patients who have a favorable response to NST, it should be noted that the studies referenced are all retrospective. Therefore, high-quality medical evidence is still needed to confirm these conclusions.

### Feasibility and safety of IR after NST

Breast reconstruction offers patients who cannot undergo BCS an opportunity for a more aesthetically pleasing breast shape and can help mitigate some of the negative effects of total mastectomy. Considerations include the benefits of immediate reconstruction (IR) versus delayed reconstruction (DR), the oncological safety of different reconstruction techniques, and the effects of combining these procedures with NST and radiation therapy.IR is associated with higher physical and psychological satisfaction compared to DR, and patients desiring reconstruction may opt for IR without compromising safety (30). Procedures such as nipple-sparing, skin-sparing, or skin-reducing mastectomies allow for IR, with nipple-sparing mastectomies requiring a negative margin at the posterior of the nipple-areola complex (31). There is a lack of high-quality evidence confirming the oncological safety of nipple-sparing mastectomy combined with reconstruction. However, several retrospective studies indicate that IR after NST does not increase the risk of local recurrence or negatively impact long-term survival. For instance, Meli et al. (32) found that there is no significant difference in local recurrence or survival between patients who undergo nipple-sparing mastectomy with or without NST, suggesting that IR is a viable and safe option. Wu et al. (33) found no significant differences in long-term outcomes including 5-year LRFS, DFS, or OS between patients who had IR after NST and those who had NR. This indicates that opting for IR does not compromise long-term survival, thus reinforcing the safety and desirability of IR. However, some caution is advised. A study by Song et al. (34) highlighted those patients with tumors exceeding 3 cm who received IR had a lower 5-year DFS compared to those who had no reconstruction, suggesting that IR may be more appropriate for smaller tumors (≤3 cm). For stage T4 breast cancer, particularly inflammatory breast cancer, Pawloski et al. (35) found that IR significantly increased the likelihood of postoperative complications and delayed the start of radiotherapy, often by more than 8 weeks. Due to these complications and the observation that the average time until the first recurrence was 18 months within a median follow-up of 4.2 years, the study recommended postponing reconstruction for at least 18 months after surgery. Wu et al. (36) reported no significant differences in LRFS, DFS, or OS between patients with poor responses to NST who underwent nipple-sparing or skin-sparing IR and those who had mastectomy alone. This suggests that the response to NST should not solely determine the choice of IR. A meta-analysis (37) of 17 studies involving 3,249 patients examined the effect of NST on postoperative complications associated IR. The analysis found that neoadjuvant NST did not significantly raise the overall risk of postoperative complications (*P* = 0.34). The analysis did show a statistically significant rise in the rate of implant or expander loss (*P* = 0.03). This suggests that while NST does not broadly elevate the risk of complications, it may specifically heighten the risk of implant-related issues.

There is widespread agreement that postmastectomy radiation therapy can lead to skin discoloration and reduction in size of the nipple-areola complex (38). In the meantime, the 2022 recommendations from the Oncoplastic Breast Consortium (39) generally agree that post-mastectomy radiation therapy (PMRT) raises the risk of complications in all forms of implant-based breast reconstruction. Most experts in the field concur that PMRT carries a lower overall long-term risk of complications after immediate autologous reconstruction compared to implant-based reconstruction. In order to avoid delaying PMRT after IR, a reverse sequence (RS) of NST, preoperative radiotherapy, mastectomy and IR has been proposed. Paillocher et al. (40) included 111 patients with RS, with a median follow-up of 31.6 months. The 5-year DFS and OS were 93.2% and 98.3%, respectively, and patient satisfaction was high (17/20). In this study, radiotherapy was feasible 4 weeks after the end of NST in the RS group, while immediate autologous latissimus dorsi breast reconstruction surgery was feasible 6–8 weeks following the conclusion of radiotherapy in the standard sequence (SS) group, and RS could shorten the treatment time. Maire et al. (41) compared the RS and SS approaches using the autologous latissimus dorsi flap with or without an implant. With a median follow-up of 61.7 months, there was no significant difference between the groups in OS (*P *= 0.44) or RFS (*P *= 0.30). Postoperative morbidity also did not differ significantly between the two groups (*P *= 0.51). In the RS group, the average time from the end of radiotherapy to surgery was 5.9 weeks, compared to 8.4 weeks in the SS group from surgery to the start of radiotherapy, indicating that RS could significantly shorten treatment time (*P *< 0.001). To further explore the optimization of treatment timelines, the ongoing single-arm clinical trial NCT05412225 (42) is investigating the feasibility of preoperative radiotherapy followed by total mastectomy and autologous IR in LABC patients. This approach aims to avoid delays in radiotherapy after IR.

For LABC patients, doctors should guide them to fully understand the process, risks and benefits of reconstructive surgery, and be clear about the expected results of surgery. Patients with original large tumor, IR should be performed with great cautiousness. T4 stage, especially inflammatory breast cancer, IR is not recommended. Patients who are willing to reconstruct and need radiation therapy can receive radiation therapy before surgery after NST to shorten the treatment time and at the same time maintain the aesthetics of the breast after reconstruction.

## Axillary lymph node management after NST

The use of NST has significantly changed the approach to axillary lymph node management in breast cancer. Traditionally, ALND was performed for patients with clinically positive nodes (cN+), but recent efforts have explored less invasive alternatives. The primary goal is to strike a balance between reducing surgical morbidity and maintaining oncological safety for these patients. A meta-analysis (43) including 33 studies revealed that axillary lymph node pCR rates by breast cancer subtypes in patients with cN+ were 60%, 45%, 48%, and 18% for HR-/HER2+, HR+/HER2+, HR-/HER2-, and HR+/HER2-, respectively. This suggests that patients with HER2+ and TNBC may be eligible for less extensive axillary surgery. Data from the Netherlands Cancer Registry (44) revealed that between 2006 and 2016, there was a notable increase in the rate of patients with initially negative axillary lymph nodes with non-invasive diagnostic methods (cN0) who underwent sentinel lymph node biopsy (SLNB) after NST, rising from 33% to 62%. Additionally, the rate of patients with cN+ who underwent ALND decreased from 99% to 63% (*P *< 0.01). There is ongoing debate about the conditions under which ALND can be safely omitted after NST. This discussion is particularly relevant when lymph nodes initially assessed as positive before treatment (cN+) are found to be negative upon pathological examination after treatment, as determined through SLNB (ypN0). The European Breast Cancer Research Association of Surgical Trialists (EUBREAST) conducted a global survey (45) in 2020, highlighting differing expert opinions on axillary management post-NST. Key points of contention include whether ALND can be omitted for patients whose positive nodes become negative (cN+→ ypN0) and the appropriate treatment for patients with sentinel lymph nodes (SLNs) showing isolated tumor cells (ypN0[i+]) or micrometastases (ypN1[mi]).

Data from large clinical trials (46–48) have found that NST potentially increase the FNR of SLNB due to its effects on axillary lymphatic reflux patterns, disruption of lymphatic structures, and induction of fibrosis. Several meta-analyses have also confirmed that the use of dual-tracer sampling and removing a minimum of three SLNs are effective in reducing FNR (49, 50). In addition, a strategy that involves marking nodes with biopsy-confirmed metastases prior to initiating NST and subsequently performing SLNB with targeted axillary dissection (TAD) has been shown to effectively reduce FNR. Anderson Cancer Center (51) revealed FNR of 10.1% and 1.4% for SLNB and SLNB in combination with TAD, respectively (*P *= 0.03).

### Safety of cN+→ ypN0 exemption from ALND

There is some controversy in the guidelines as to whether cN+→ypN0 patients should be spared from ALND. Barrio et al. (52) conducted an analysis on a cohort of 610 patients diagnosed with cN1. Among those patients, 91% who were ypN0 underwent SLNB alone. It was found that 42% of these patients had three or more SLNs removed, and 70% of them received regional nodal irradiation (RNI). At a median follow-up of 40 months, recurrence in the axillary nodes was noted in just one patient, who did not undergo RNI. This study suggested that cN1→ ypN0 patients, and who had three or more SLNs identified through SLNB, may not require ALND. Tinterri et al. (53) studied 291 patients who were ypN0 after SLNB, including 131 who were cN0 and 160 who were cN+ before treatment. After a median follow-up of 43 months, the local recurrence rates in the axillary nodes were 2.3% for patients with cN0 and 1.3% for those with cN+. There were no significant differences in DFS and OS between the cN0 and cN+ groups or between those who had SLNB and those who had ALND. Similarly, Kahler et al. (54) analyzed 688 ypN0 patients after SLNB, with a median follow-up of 9.2 years. They observed local axillary recurrence rates of 1.8% for cN0 patients and 1.5% for cN1-2 patients, with no significant difference in DFS and OS between the groups. These retrospective studies consistently show that cN+ → ypN0 patients do not necessarily need ALND. However, some limitations exist, such as Tinterri et al.'s lack of detailed information about cN+ patients and Kahler et al.'s inclusion of only 12 cases of cN2 patients. In contrast, Park et al. (55) analyzed data from 22,156 cN2-3 patients in the National Cancer Database. Of these, 2,190 (9.9%) underwent SLNB. After adjusting for relevant factors, the study found that ALND was linked to a reduced risk of mortality compared to SLNB, even in patients who achieved pCR. In a study conducted by Lim et al. (56), 477 patients with cN1→ ypN0 were analyzed. At a median follow-up of 65 months, patients who underwent ALND had worse DFS (*P *= 0.011) and OS (*P *= 0.0476) compared to those who had only SLNB. They noted that the ALND group had a higher number of patients with larger tumors (T3-4). However, in the subgroup of patients with smaller tumors (cT1–2), there was no significant difference in DFS and OS between the two groups. The details of the corresponding retrospective studies are provided in Table 1.

**Table 1:** Summary of retrospective studies on cN+→ ypN0 patients.

Study	Enrollment	Surgical technique	Follow-up	Primary outcome
Barrio et al. (52)	555cN1	234 SLNB 321 ALND (no ypN0 or <3 negative SLNs)	40 months	Nodal recurrence:1(refuse NRT).
Tinterri et al. (53)	131 cN0 160 cN+	226 ypN0: SLNB 65 ypN0: ALND	43 months	No statistically differences in DFS、OS between cN0 and cN+ nor between SLNB only or ALND.
Kahler et al. (54)	466 cN0 211 cN1 11 cN2	428 ypN0: SLNB 40 ypN+: SLNB 220 ypN+: ALND	9.2 years	Among 428 ypN0 patients, LRFS、OS、DFS has no difference between cN0 and cN1/2 groups.
Park et al. (55)	15176 cN2 6979 cN3	2190 SLNB 19966 ALND		ALND was associated with improved survival (*p* < 0.001) even for patients who achieved ypN0.
Lim et al. (56)	477 cN1	314 SLNB (17.5% cT3-4) 163 ALND (27.6% cT3-4)	65 months	ALND patients had significantly worse DFS (*P* = 0.011) and OS (*P* = 0.0476) but not in the cT1-2 group.

The AXSANA trial (57), a multi-center prospective study, aims to recruit a total of 3,000 patients by the year 2030. The aim of this study is to evaluate the feasibility and safety of various surgical techniques, including ALND, SLNB, and TAD, in patients with positive lymph nodes. In summary, for LABC patients, particularly those achieving ypN0 status, it may be appropriate to consider less invasive surgical options like SLNB, especially when dealing with smaller tumors and fewer affected lymph nodes. However, for patients with more extensive disease, ALND may still be warranted until the final results of the AXSANA trial are available.

### Axillary lymph node management for ypN0(i+) and ypN1(mi)

The management of ypN0(i+) or ypN1(mi) remains a topic of debate. Wong et al. (58) showed that the 5-year DFS of ypN0(i+) and ypN1(mi), ypN0 patients in the Dana-Farber/Brigham and Women's Cancer Center (DFBWCC) was 73.5%, 74.7%, and 88.4% (*P* < 0.001); the 5-year OS in the NCDB database was 82. 8%, 79.5%, and 88.9% (*P* < 0.001). Subgroup analyses indicated that ypN0(i+) and ypN1(mi) exhibited a poorer prognosis compared to ypN0, particularly in cases of HER2+ and TNBC. Pending results from large clinical trials, this study suggested that patients with ypN0(i+) and ypN1(mi) should undergo ALND. In contrast, a retrospective study (59) conducted in the Netherlands revealed that there was no difference in the 5-year OS (*P *= 0.889) or DFS (*P *= 0.613) rates between patients with ypN0(i+) and ypN1(mi) compared to those with ypN0. The potential explanation for this disparity, aside from population variation, could be attributed to the fact that 70.4% of ypN0(i+) and 80.3% of ypN1(mi) cases included in the DFBWCC study ultimately underwent ALND, whereas in the Netherlands study, all patients underwent ALND. Kantor et al. (60) analyzed 4,496 patients with HR+/HER2-, finding no statistically difference between ypN0 or ypN+ in LRFS, OS, and DFS between those who underwent ALND and patients who did not. Based on these findings, the study suggested that ypN0(i+) and ypN1(mi) patients may not need ALND after neoadjuvant endocrine therapy. The international multicenter retrospective OPBC-05/ICARO study (61) examined 583 patients with ypN0(i+). Among them, 182 patients received ALND, while 401 did not. Among those who underwent ALND, 30% were found to have additional positive nodes. There was no significant difference in the 5-year rate of any oncologic outcomes. Consequently, the study suggests that routine ALND may not be necessary for this patient population. Existing studies present conflicting findings regarding the potential exemption of ypN0(i+) and ypN1(mi) from ALND. In the EUBREAST survey (45), it was found that 32.3% of the experts recommended no additional treatment for ypN0(i+) patients, while 33.1% suggested RNI. A total of 34.8% of the experts recommended ALND for ypN1(mi) patients, while 30.4% expressed a preference for RNI. The prevailing viewpoint among experts at the 2021 St. Gallen Conference (22) was in favor of RNI as opposed to ALND for patients with ypN0(i+), ypN1(mi). However, definitive guidelines are pending the results of ongoing clinical trials. The OT1-3-02 (62) and NSABP B-51/RTOG 1,304 (63) clinical trials were conducted to enroll patients with ypN0(i+) in order to examine the potential benefits of RNI. The Alliance A11202 (64) and TAXIS (65) phase III trials are evaluating the safety of omitting ALND in patients with ypN1(mi). The details of the corresponding retrospective studies are provided in Table 2.

**Table 2:** Summary of retrospective studies on ypN0(i+) and ypN1(mi) patients.

Study	Enrollment	Surgical technique	Follow-up	Primary outcome
Wong et al. (58)	967 cT1-4 N0-1	524 ypN0 (37.6%ALND) 27 ypN0(i+) (70.4%ALND) 61 ypN1(mi) (80.3%ALND) 221ypN1 (94.1%ALND) 134 ypN2-3 (100%ALND)	5 years	ypN0(i+) and ypN1(mi) exhibited a poorer prognosis compared to ypN0 and ALND is recommended.
Nijnatten et al. (59)	1347 cN+	299 ypN0 (SLNB) 51 ypNi/mi (ALND) 997 ypN1-3 (ALND)	5 years	No differences in DFS、OS between ypN0(i+) and ypN1(mi) compared to ypN0.
Kantor et al. (60)	4495 cT1-3 N0-1 (HR+/HER2-)	2510 ypN0 99 ypN0(i+) 257ypN1(mi) 948ypN+ (32.1% ALND)	5 years	No difference between ypN0 or ypN+ in LRFS, OS, and DFS between those who underwent ALND and patients who did not. ALND can be avoided.

### Possibility of ALND omission in ypN+ patients

ALND is commonly used as a standard treatment for breast cancer patients with positive SLNs, but it is associated with significant morbidity. Recently, there has been interest in finding less invasive alternatives that maintain oncological safety. Efforts have been made to investigate the potential of SLNB and RNI as a safe alternative to ALND in certain cases. The ACOSOG Z0011 study (66) included 892 female patients with T1 or T2 breast cancer who underwent BCS and had metastases in one or two SLNs without palpable axillary lymphadenopathy, were followed for a median of 9.3 years. The results indicated that SLN alone was not inferior to that of patients treated with ALND. The AMAROS Trial (67) included 1,425 patients with cT1-2, node-negative breast cancer and a positive sentinel node biopsy, who were randomly assigned to either ALND or NRT. The 10-year analysis shows that both treatments resulted in a low axillary recurrence rate, with no significant differences in OS, DFS, or locoregional control. Though these two clinical trials did not include patients who received NST, they still provide a potential option for patients with LABC. Efforts have been made to find less invasive procedures for ypN+ patients.

The retrospective study conducted by Almahariq et al. (68) included patients with cT1-3N1 who were converted to ypN1 after NST from the NCDB. Out of the total sample, 1,313 patients underwent ALND and 304 patients underwent SLNB. All patients received RNI. The study found a statistically significant difference in 5-year OS between the two groups, with a higher survival rate in the ALND group compared to the SLNB (*P* = 0.01). Furthermore, the multivariate analysis revealed that SLNB was linked to lower survival rates (*P* < 0.001). A different conclusion was reached by Park et al. (69), who conducted a study incorporating data from 14 medical centers in South Korea. The study included 1,103 cases of ALND and 170 cases of SLNB, with all patients receiving RNI. With a median follow-up period of 75.3 months, the study found no statistically significant disparity in DFS (*P* = 0.406) or OS (*P* = 0.083) between two groups, and multivariate analysis indicated SLNB did not compromise oncological outcomes, suggesting that exemption from ALND could be a feasible option for ypN+ patients receiving RNI. The study conducted by Almahariq et al. (68) encountered limitations in data extraction from the NCDB, resulting in the specification of SLNs ranging from 1 to 4. On the other hand, the study conducted by Park et al. (69) did not impose any restrictions on the number of positive lymph nodes either prior to or following NST, but the median number of SLNs in this study was 6. A study conducted by Moo et al. (70) involving 273 patients with positive SLNs undergoing ALND revealed a high incidence of ALND positivity across all molecular subtypes, with no significant difference observed between micrometastases and macrometastases. Therefore, they recommend performing ALND for patients with positive SLNs, regardless of molecular subtype.

Several ongoing trials are exploring these alternatives. Alliance A11202 (64) is an ongoing phase III clinical trial to enroll 2012 cN1 patients with positive SLNs after NST, with one group receiving ALND followed by RNI and the other group receiving RNI alone. TAXIS (65) is a multinational, multicenter phase III clinical trial aimed at assessing the viability and effectiveness of exempting cN1-2 patients with positive SLNs from ALND, which includes both patients who receive NST or not. ADARNAT (71) is a multicenter, randomized, open-label phase 3 trial involving 1,660 patients with 1-2positive SLNs post-NST, across 50 Spanish centers. Patients will be assigned at random to either a group receiving NRT without ALND or a group undergoing ALND. The primary outcome is 5-year axillary recurrence. Table 3 provides detailed information on all the ongoing trials mentioned in this section.

**Table 3:** Summary of ongoing large clinical trials.

Trial	Enrollment	Aim and procedures	Follow-up	Outcome measures
AXSAN (57)	cT1-4 N+→ ycN0（SLNB、 TAD、ALND）	To evaluate SLNB、 TAD、ALND in cN+ patients treated With NST.	5years	invasive DFS, axillary recurrence rate, health-related quality of life, arm morbidity
Alliance A11202 (64)	cT1-4 N1 →ypN+(ypN0[i+] excluded) (SLNB, SLNs:1-8)	Assess the safety and outcomes of omitting ALND in patients who have ypN+ in the SLNB after NST Arm 1: ALND + NRT Arm 2: NRT + ART	8 years	IBC-RFI, OS, ILR-REC, Arm morbidity, Breast lymphedema
OT1-3-02 (62)	cT1-3 N1→ypN0 (SLNB、ALND)	To determine if NRT can reduce the recurrence of cN1→ypN0 patients. Arm 1: NRT Arm 2: no NRT	7.5 years	IBC-RFI, OS, LRRFI, DRFI, DFS-DCIS
NSABP-B-51 (63)	cT1-3N1→ypN0 (SLNB、ALND)	Evaluating NRT in cN1→ypN0 patients after NST. Arm 1: no NRT Arm 2: NRT	10 years	IBC-RFI, OS, LRRFI, DRFI, DFS-DCIS, Time to SPC
TAXIS (65)	cT1-3 N1-3→ypN+ (tailored axillary surgery, ypN0[i+] included)	Tailored axillary surgery and RT is non-inferior to ALND in terms of DFS of node positive patients at high risk of recurrence. Arm 1: ALND + NRT Arm 2: NRT + ART	20 years	OS, BCSS, STTLR, TTDR, Lymphedema, Shoulder motion
ADARNAT (71)	cT1-4N0-1→ypN+ (SLNB, 1–2 positive nodes)	To evaluate whether NRT is non-inferior to ALND in terms of 5-year axillary recurrence Arm 1: NRT Arm 2: ALND	5 years	Axillary locoregional recurrence, DFS, OS, Quality of life, Lymphedema

BCSS, breast cancer-specific survival; TTLR, time to local recurrence; TTDR, time to distant recurrence; IBC-RFI, invasive breast cancer—recurrence-free interval; DRFI, Distant recurrence-free interval; DFS-DCIS, disease-free survival for ductal carcinoma *in situ*; SPC, second primary cancer; STTLR survival time to local recurrence; ART, axillary radiation therapy.

## Discussion

In conclusion, the management of LABC remains complex, requiring tailored to individual patient profiles. Challenges still persist in predicting complete responses to NST and omitting surgery seems inappropriate for LABC patients at this time. Existing retrospective studies have demonstrated the safety of BCS in patients who respond well to NST. However, the standard for negative margins after NST needs to be further validated through large-scale randomized controlled trials. For LABC patients with a desire for reconstruction, IR does not compromise tumor safety and does not increase complications. Patients who have large initial tumors should be cautious, and IR at T4 is not recommended. Patients with a desire for IR who still need radiotherapy after surgery, a reverse treatment sequence of NST, preoperative radiotherapy, mastectomy, and IR is feasible. Regarding axillary lymph node management, guidelines emphasize the importance of dual tracer imaging and the identification of three or more SLNs due to the increased FNR following NST. Some of the available retrospective studies had SLNs less than 3, which could potentially contribute to the significant variability in the results. Therefore, it is imperative to include SLN ≥3 as a crucial criterion in the design of clinical trials that explore exemptions from ALND. Based on the findings of retrospective studies, patients who have cN0-1 →ypN0 can potentially be excluded from undergoing ALND. However, it takes extreme caution when treating patients with cN2-3 →ypN0, and ALND is strongly recommended in such cases. As to whether the presence of micrometastases or macrometastases in the SLNs can exempt patients from ALND, the results of the available studies are inconsistent and definitive conclusions will have to await the long-term survival data from the various ongoing clinical trials. We have observed that few clinical studies on axillary lymph node management have considered the impact of different molecular subtypes. As noted by Swarnkar (72), persistent positive lymph nodes after treatment may suggest a more aggressive tumor in HER2-positive and TNBC patients, as these subtypes are generally more responsive to NST compared to luminal subtypes. This observation implies that such cases might necessitate more aggressive surgical approaches, such as ALND. Additional research is required to confirm this hypothesis and to further refine treatment strategies. Overall, the surgical approach following NST for LABC should be tailored based on pre-treatment clinical characteristics, NST efficacy, and the patient's overall condition. A balanced consideration of the benefits and risks, aligned with the patient's preferences, should guide a collaborative decision between the patient and the surgeon.
